# In vivo quantification of ^177^Lu with planar whole-body and SPECT/CT gamma camera imaging

**DOI:** 10.1186/s40658-015-0123-2

**Published:** 2015-09-17

**Authors:** Dale L. Bailey, Thomas M. Hennessy, Kathy P. Willowson, E. Courtney Henry, David L.H. Chan, Alireza Aslani, Paul J. Roach

**Affiliations:** Department of Nuclear Medicine, Royal North Shore Hospital, St Leonards, NSW 2065 Australia; Faculty of Health Sciences, University of Sydney, Cumberland, NSW Australia; Institute of Medical Physics, University of Sydney, Camperdown, NSW Australia; Sydney Medical School, University of Sydney, Camperdown, NSW Australia; NETwork, Sydney Vital, St Leonards, Sydney, NSW Australia

## Abstract

**Background:**

Advances in gamma camera technology and the emergence of a number of new theranostic radiopharmaceutical pairings have re-awakened interest in in vivo quantification with single-photon-emitting radionuclides. We have implemented and validated methodology to provide quantitative imaging of ^177^Lu for 2D whole-body planar studies and for 3D tomographic imaging with single-photon emission computed tomography (SPECT)/CT.

**Methods:**

Whole-body planar scans were performed on subjects to whom a known amount of [^177^Lu]-DOTA-octreotate had been administered for therapy. The total radioactivity estimated from the images was compared with the known amount of the radionuclide therapy administered. In separate studies, venous blood samples were withdrawn from subjects after administration of [^177^Lu]-DOTA-octreotate while a SPECT acquisition was in progress and the concentration of the radionuclide in the venous blood sample compared with that estimated from large blood pool structures in the SPECT reconstruction. The total radioactivity contained within an internal SPECT calibration standard was also assessed.

**Results:**

In the whole-body planar scans (*n* = 28), the estimated total body radioactivity was accurate to within +4.6 ± 5.9 % (range −17.1 to +11.2 %) of the correct value. In the SPECT reconstructions (*n* = 12), the radioactivity concentration in the cardiac blood pool was accurate to within −4.0 ± 7.8 % (range −16.1 to +7.5 %) of the true value and the internal standard measurements (*n* = 89) were within 2.0 ± 8.5 % (range −16.3 to +24.2 %) of the known amount of radioactivity contained.

**Conclusions:**

In our hands, state-of-the-art hybrid SPECT/CT gamma cameras were able to provide accurate estimates of in vivo radioactivity to better than, on average, ±10 % for use in biodistribution and radionuclide dosimetry calculations.

## Background

The gamma camera is the workhorse of nuclear medicine imaging. However, the majority of clinical studies performed using the gamma camera today are used qualitatively and, as such, are not primarily aimed at accurately quantifying the in vivo radiopharmaceutical distribution. Positron emission tomography (PET) in contrast is, however, regarded as being a quantitative imaging modality that, in addition to providing images of the distribution of positron-emitting radionuclides in the body, allows measurements of radionuclide uptake in organs and tissues (e.g. standardised uptake value (SUV)) for a variety of reasons such as attempting to determine the metabolic activity of a tumour, prognostication or assessment of response to chemotherapy or radiotherapy. While the gamma camera is frequently used to measure functional parameters from organs, such as renal function, left ventricular ejection fraction, relative myocardial perfusion and thyroid uptake, the data obtained in these settings are non-quantitative in that the analyses are based on the count rates of the gamma rays recorded by the detector and not normally converted to absolute values of radioactivity concentration (e.g. kBq mL^−1^). The reasons for this are largely historical with PET being regarded as the only quantitative imaging modality available using radiolabelled tracers.

In recent times, however, this situation has started to change. There are numerous reasons why quantitative measurements with the gamma camera are being re-visited. These include:

- The developments in the past 5–10 years of gamma cameras incorporating X-ray CT scanners and improved reconstruction algorithms for emission tomography with enhanced corrections for the attenuation and scattering of the gamma rays as they transit the body, leading to accurate, quantitative cross-sectional single-photon emission computed tomography (SPECT) imaging with a variety of radionuclides
- Advances in radiochemistry resulting in a new range of radionuclide therapies, many of which co-emit gamma rays as well as beta particles, thereby facilitating the temporal measurement by gamma camera imaging of the whole-body biodistribution and pharmacokinetics
- Re-evaluation of existing clinical studies using single-photon radionuclides that may benefit from a more quantitative assessment. Examples proposed include measuring myocardial blood flow and fractional flow reserve and SUVs in ^99m^Tc bone scans for monitoring response to therapy [1]
- The desire to individualise radionuclide therapies for cancer subjects based on their observed response to tracer doses to better protect critical organs such as the bone marrow and kidneys while optimising the absorbed radiation dose delivered to the tumours

In previous publications, we have reported our methodology for CT-based quantification in SPECT using ^99m^Tc and ^201^Tl, which we refer to as qSPECT [2, 3]. The aim of this paper is to report on the extension of these methods to accurately estimate lutetium-177 (^177^Lu) radioactivity in vivo with SPECT/CT as well as applying previously published methods to quantify 2D whole-body planar images, due the high level of recent interest in ^177^Lu for radionuclide therapy applications. A previous publication from a different group on quantitative ^177^Lu SPECT, which used the manufacturer’s provided software for all calculations, showed that accuracy of approximately ±5 % could be achieved [4].

## Methods

### Gamma camera

The gamma cameras that have been used in this work are all hybrid SPECT/CT devices. The models that have been used are Symbia T6 and Intevo (both from Siemens Healthcare, Hoffman Estates, IL, USA). Both systems have a 15.8-mm-thick scintillation crystal for improved efficiency of detection of higher energy γ photons since much of the routine work on these cameras involves ^131^I (*E*_γ_ = 364 keV), ^67^Ga (93, 185 and 300 keV), ^111^In (171 and 245 keV) and, increasingly, ^177^Lu (208 keV). Both systems are dual-head detector systems and incorporate a six-slice X-ray CT scanner. All imaging used medium energy collimators and a single pulse height analyser (PHA) gamma energy photopeak centred on the 208-keV photon emitted by ^177^Lu. While the abundance of this particular photon is only 11 % of all disintegrations, when using therapeutic amounts involving up to 10 GBq of radionuclide, the photon flux is sufficient for adequate imaging to be performed. For this reason, we have not investigated imaging other photons emitted by ^177^Lu with multiple energy windows. Both systems would be regarded as state-of-the-art with the Intevo, in particular, having enhanced reconstruction algorithms aimed at quantitative SPECT imaging (although not yet implemented for ^177^Lu imaging).

### ^177^Lu

The ^177^Lu (*t*_½_ = 6.7 days) that has been used in this work was produced in a nuclear reactor by an (n, γ) reaction (ITG, Isotope Technologies Garching GmbH, Germany and the Australian Nuclear Science and Technology Organisation (ANSTO), Australia). Ytterbium-176 (^176^Yb) is irradiated to produce ^177^Yb which decays with a half-life of 1.9 h to ^177^Lu. This method of production produces “no carrier added” (nca) ^177^Lu as the irradiated ytterbium target is readily separated chemically from the lutetium daughter product. An alternative method to make ^177^Lu using the ^176^Lu (n, γ) ^177^Lu reaction will contain other isotopes of lutetium (^176^Lu, ^177m^Lu) as contaminants in the final product.

### 2D quantitative planar imaging

Planar imaging was used to measure the whole-body clearance and retention of the nca ^177^Lu over a number of days. We have acquired these data routinely when treating neuroendocrine tumours (NETs) which express somatostatin receptors using radionuclide therapy (RNT) with the chelated peptide referred to as DOTATATE (DOTA^0^-(Tyr^3^)-octreotate where DOTA = 1,4,7,10-tetra-azacyclododecane–1,4,7,10-tetraacetic acid) (Auspep, Melbourne, Australia). In the work reported in this paper, all preparations of nca [^177^Lu]-DOTATATE followed the methods described in a recent paper from our institution by Aslani et al. [5] and were accompanied by a 3-h amino acid infusion in order to provide protection for the kidneys from radiation by preventing re-absorption of the [^177^Lu]-DOTATATE in the proximal tubules and hence enhancing clearance from the kidney to the bladder. All imaging used standard acquisition software without modification to the gamma camera, scanning bed or other hardware. The whole-body scanning speeds varied between 10 and 20 cm min^−1^ depending on the expected count rates from the subject on each day of imaging leading to total whole-body scanning times of between 10 and 20 min.

To produce quantitative planar images, we have followed the methodology originally described in MIRD Pamphlet #16 [6] and implemented using in-house developed software using a high-level scientific programming language (IDL, Exelis Visual Information Solutions, Herndon, VA, USA) on a dedicated nuclear medicine workstation (HERMES, Nuclear Diagnostics, Stockholm, Sweden). A radionuclide transmission scan was used for attenuation correction. This used a ^57^Co sheet source normally used for quality assurance to collect a pair of scans: the first with the transmission source placed on the face of one detector irradiating the opposite detector in whole-body mode to measure the unattenuated photon flux from the source (usually referred to as a “blank” or “reference” scan), and the second with the identical set-up but with the subject lying on the scanning bed prior to the injection of the radiopharmaceutical. A small flask containing non-radioactive water was placed at the top of the field of view. A radioactive version of an identical flask containing the same volume of water with ^177^Lu added was used in all scans as an internal standard to calibrate the system and for quality assurance. Care was taken to note the positioning of the subject so that this could be reproduced accurately at each time point when whole-body planar imaging was acquired. The subjects were then administered their radionuclide therapy over approximately 20 min.

All planar whole-body scans after the administration of the radionuclide therapy contained the flask with a known amount (~20 MBq) of ^177^Lu for the internal calibration. The count rate measured over time from the flask was also used to assess the observed decay of the source and whether this agreed with the known value for the half-life of ^177^Lu. In this way, any effects such as changes in the dead time of the detector system, variations in the scanning bed speed or background contamination would likely be observed. Both detector heads were used to acquire the subject data so that a geometric mean (GM) whole-body planar image was produced during the analysis, as described in the MIRD methodology.

**Fig. 1:**
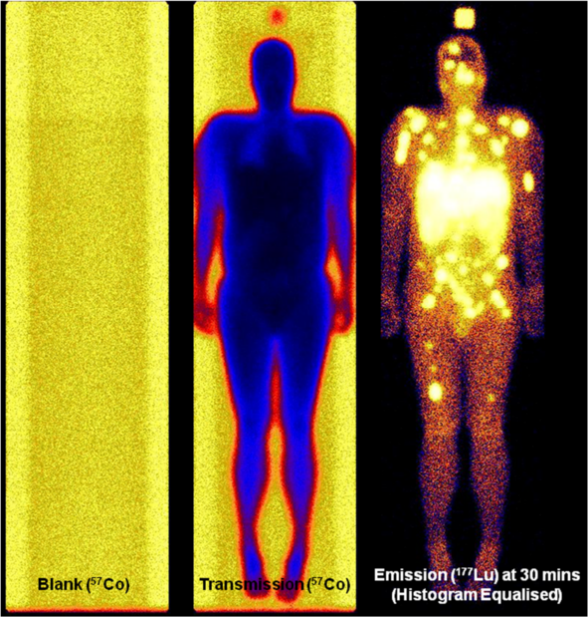
Planar whole-body scans from one individual for the blank (*left*) and transmission scans (*middle*) both using a ^57^Co sheet source and the initial geometric mean whole-body emission scan commencing approximately 30 min after the [177Lu]-DOTATATE infusion (*right*) are shown. Due to the very high contrast in the emission scan, it has been histogram normalised to more effectively display the full range of the data. Numerous metastatic lesions (*yellow spots*) are seen in the emission image. The calibration source is clearly visible above the head of the subject

The software that was used to produce the quantitative whole-body planar images was developed in-house in IDL. The radioactivity estimated in the *j*th pixel in the GM images from [6] is given by

$$ {A}_j=\sqrt{\frac{I_A{I}_P}{e^{-{\mu}_et}}}\frac{f_j}{C} $$

where *A*_*j*_ is the radioactivity (in kBq) in the *j*th pixel, *I*_*A*_ and *I*_*B*_ are the anterior and posterior count rates for the *j*th pixel derived from the images, the exponential term (−*μ*_*e*_*t*) is the attenuation measured from the ratio of the reference-to-transmission scan, *f*_*j*_ is a term which accounts for the source self-attenuation [6] and *C* is a calibration factor (cps kBq^−1^) derived from the internal standard. An adjustment for the difference between the energy of the gamma photons from ^57^Co (122 keV) to ^177^Lu (208 keV) is required (see Table 1). This conjugate view GM technique allows determination of activity within the volume of interest without requiring knowledge of the depth of the source region and without dependence upon assumptions inherent in single-view imaging. At the conclusion of processing, the images are stored with units of kilobecquerel in such a way that the total number of events in the image is equal to the radioactivity in standard units (kBq). Figure 1 shows an example of the data that are used in this process. Note that unlike many investigations using radionuclides, decay correction is not applied to these time series data as the radioactivity present within the body provides the therapeutic effect and, as such, the total radioactivity present at the time of imaging is the desired parameter. It would be appropriate to apply decay correction to a study where, for example, the biodistribution and whole-body retention of a radiolabelled therapeutic pharmaceutical were the parameter of interest. This is not, however, the case for radionuclide therapies.

**Table 1:** Parameters used in the processing of the quantitative planar and SPECT data

Imaging mode	Parameter	Value	Units	Comment
Planar	Attenuation coefficient in water: ^57^Co	0.125	cm^−1^	Used in scaling transmission scan; broad-beam value
Planar	Attenuation coefficient in water: ^177^Lu	0.090	cm^−1^	Used in scaling transmission scan; broad-beam value
SPECT	Transmission-dependent scatter correction parameter: *A*	2.15	–	Constant used in scatter correction of ^177^Lu data
SPECT	Transmission-dependent scatter correction parameter: *B*	1.15	–	Constant used in scatter correction of ^177^Lu data
SPECT	Transmission-dependent scatter correction parameter: *β*	0.29	–	Constant used in scatter correction of ^177^Lu data
SPECT	FWMH of Gaussian in transmission-dependent scatter correction kernel	0.344	cm^−1^	Constant used in scatter correction of ^177^Lu data
SPECT	Attenuation coefficient in water: ^177^Lu	0.110	cm^−1^	Used for attenuation correction of SPECT; narrow-beam value

### 3D quantitative SPECT imaging

The methodology that we have developed and previously reported for quantitative SPECT [2, 7–9] has been adapted for imaging the ^177^Lu distribution in vivo, with appropriate modifications for the radionuclide. This uses the CT scan with conversion from Hounsfield units to attenuation coefficients for attenuation correction and for transmission-dependent scatter correction [10]. An iterative reconstruction algorithm (ordered subset estimation maximisation (OSEM)) [11] was used for the SPECT image reconstruction. This methodology, while originally developed for diagnostic studies, is well-suited to imaging therapeutic levels of ^177^Lu in vivo as, in spite of the relatively low gamma photon abundance for the 208-keV photon from ^177^Lu, the amount administered (~GBq) readily lend these studies to producing images of high quality with a satisfactory event rate for detection. The event rates detected in the initial few days of imaging resemble those from a diagnostic ^99m^Tc bone scan. The imaging characteristics of ^177^Lu were assessed in a series of phantom experiments, as we have done for other radionuclides, to derive parameters such as system sensitivity and the a priori constants (*A*, *B*, *β*, *μ*) used in the scatter and attenuation correction procedures. These are contained in Table 1. Apart from these different optimising parameters, the methodology that we have previously published was unchanged. The images thus produced display the data as a concentration unit (kBq mL^−1^) per voxel.

To validate the methodology in the reconstructed clinical images, we have used two approaches: (a) all SPECT acquisitions included a plastic flask containing water (~125 mL) with a calibrated amount of ^177^Lu added (~40 MBq) that acted as an internal standard to check the accuracy of the reconstruction and (b) an in vivo approach similar to that which we have previously employed with blood pool imaging, using the concentration of the radionuclide in a venous blood sample taken at approximately the mid-point of the SPECT acquisition to compare to the reconstructed concentration of radioactivity in the images [7].

### Imaging protocol

The imaging protocol we have used originally acquired planar whole-body images commencing immediately at the end of the radionuclide therapy infusion (~30 min after the infusion commenced) and again at approximately 4, 24 and 96 h post-infusion. These were supplemented with SPECT/CT scans of the upper abdomen at the 4, 24 and 96 h time points. In the SPECT scans, the time per projection was initially 10 s for 60 views/detector leading to a total imaging time of approximately 10–12 min. This was increased in the latter imaging time points initially to 15 s per projection and then 20 s per projection, thus requiring a little over 15–20 min per scan, respectively. After acquiring a large number of planar whole-body scans (~30) at the early imaging time point, we discontinued this and replaced it with a SPECT/CT scan immediately after the infusion ceased. During this scan, a blood sample was taken for determining the radioactivity concentration in whole blood with an automated laboratory bench-top gamma counter. The technical parameters for the protocol are contained in Table 2. All subjects were treated as outpatients not requiring hospitalisation and returned to their homes or current lodgings at the end of each day.

**Table 2:** Acquisition parameters for all time points. A medium energy collimator and a single PHA energy window of 208 keV ± 10 % were used for all acquisitions

Approx. time from injection (h)	Cohort	Acquisition	Parameters	Comments
Morning of RNT	Both	Blank planar WB scan	^57^Co sheet source; 10 cm min^−1^; range 0–199 cm; matrix 1024 × 256	
−1	Both	Transmission planar WB scan	^57^Co sheet source; 10 cm min^−1^; range 0–199 cm; matrix 1024 × 256	Prior to administration of [^177^Lu]-DOTATATE
0.5	1	Whole-body planar emission scan	^177^Lu; 20 cm min^−1^; matrix 1024 × 256	Prior to voiding—100 % of administered radioactivity remaining in subject
0.5	2	SPECT/CT of the thorax and abdomen	^177^Lu; 10 s/projection, 120 projections (3° radial sampling), step and shoot mode; acquisition matrix 128 × 128	Venous blood sample taken at mid-point of scan
~4	Both	Whole-body planar emission	^177^Lu; 20 cm min^−1^; matrix 1024 × 256	
~4	Both	SPECT/CT over the kidneys	^177^Lu; 15 s/projection, 120 projections (3° radial sampling), step and shoot mode; acquisition matrix 128 × 128	Time per projection increased due to lower retained radioactivity
~24	Both	Whole-body planar emission	^177^Lu; 10 cm min^−1^; matrix 1024 × 256	
~24	Both	SPECT/CT over the kidneys	^177^Lu; 20 s/projection, 120 projections (3° radial sampling), step and shoot mode; acquisition matrix 128 × 128	Time per projection increased to reflect decreased overall retained radioactivity
~96 or 120	Both	Whole-body planar emission	^177^Lu; 10 cm min^−1^; matrix 1024 × 256	
~96 or 120	Both	SPECT/CT over the kidneys	^177^Lu; 20 s/projection, 120 projections (3° radial sampling), step and shoot mode; acquisition matrix 128 × 128	

The imaging protocol described was repeated for all cycles of radionuclide therapy for both cohorts. In this way, we were able to assess the accuracy and reproducibility of the whole-body clearance, retention and uptake of the radionuclide administered for therapy. A summary of the imaging acquisition protocols is contained in Table 2.

### Subject details

#### Cohort 1

In the initial series of scans (cohort 1), we acquired planar whole-body (anterior/posterior) images of the subjects immediately after the end of the [^177^Lu]-DOTATATE infusion prior to voiding. Thus, by accurately knowing the amount of ^177^Lu that was administered from the dose calibrator measurements before and after the injection, we were able to compare the known total amount of radioactivity contained in the subject’s body with that estimated from the planar gamma camera images.

#### Cohort 2

A second group of subjects had SPECT/CT scans acquired immediately at the conclusion of the [^177^Lu]-DOTATATE infusion, but in this case, they were allowed to empty their bladder prior to commencing the data acquisition. The study commenced approximately 40 min after the start of the infusion, chosen as this is a time when the concentration of [^177^Lu]-DOTATATE in the blood is still reasonably high. The same procedure was initially attempted at the 4-h time point during SPECT/CT acquisition; however, the clearance from the blood was so rapid in most subjects that it was difficult to reliably measure the blood concentration of ^177^Lu in the SPECT images at this time. Therefore, in spite of the fact that the rate of clearance is rapid and hence the change in concentration in the blood during the acquisition is likely to be a confounding factor, it was decided to measure the blood pool early to give the best possible image quality for the measurements.

Venous blood was withdrawn from the cannula that was being used for the amino acid infusion, and was taken at approximately the mid-point of the SPECT acquisition; the acquisition was suspended temporarily if required for the sample to be taken. Saponin (Sigma-Aldrich, Sydney, Australia) was added to the blood sample to lyse the red cells to produce a homogeneous mixture. Half millilitre aliquots of the lysed whole blood were transferred by pipette to counting tubes for the automatic gamma counter (Wizard^2^ Automatic Gamma Counter 2480, PerkinElmer, Waltham, MA, USA) and counted with a ^177^Lu standard prepared on each day of therapy. The samples were generally counted within 4–24 h after the blood sample was taken.

### Data analysis

In cohort 1, the image-estimated total radioactivity at the initial imaging time point was compared with the net amount administered to the subject based on measurements in a dose calibrator of the syringe containing the [^177^Lu]-DOTATATE before and after the administration of the radionuclide therapy. No account for retained radioactivity in the infusion lines and cannula was included. Due to the way that the software has been written, the total radioactivity in the geometric mean whole-body planar images is equivalent to the total number of events (“counts”) in the image. The difference between the known amount of therapy administered and the image estimate was calculated for each time point as:

$$ \mathrm{Difference}\left(\%\right)=\frac{\left(\mathrm{image}\ \mathrm{estimate}\ \left(\mathrm{MBq}\right)-\mathrm{calibrated}\ \mathrm{dose}\ \left(\mathrm{MBq}\right)\right)}{\mathrm{calibrated}\;\mathrm{dose}\;\left(\mathrm{MBq}\right)}\times 100 $$

Thus, a positive value for the difference indicates an *over*estimate in the whole-body image while a negative value indicates an *under*estimate of the amount of radioactivity in the body.

In cohort 2, the blood sample measured in the gamma counter was compared to a standard made up on the day to convert counts per minute (cpm) from the counter to kilobecquerel per millilitre and decay corrected from the time of counting back to the mid-point of the SPECT acquisition. The blood pool concentration was measured in the reconstructed images with the voxel values displayed in units of kilobecquerel per millilitre. The data over five contiguous planes were averaged to improve the signal to noise ratio. A circular ROI was placed over the middle of the thoracic vasculature (cardiac blood pool), and all pixels above 80 % of the maximum value within the region were used as a mask to identify the vascular structures. The mean value and standard deviation within these pixels were recorded and an overall mean and standard deviation of the means calculated.

In the SPECT studies containing the internal standard (cohorts 1 and 2), the total radioactivity contained within the flask was estimated from the images by placing a generous, or “loose”, region of interest (ROI) around the flask on a representative transverse slice with the reconstructed voxel values summed over all slices containing the flask. The known amount of ^177^Lu contained in the flask was calculated with a dose calibrator by measuring the difference between the amount contained in the dispensing syringe before and after it was added to the flask. A new calibration standard was made up for each initial day of imaging, but only one standard was used for all subjects treated on the same day and in all subsequent imaging (i.e. up to day 4 or 5). The results are expressed as the total radioactivity estimated in the flask from the images compared to the calibrated amount dispensed.

## Results

### 2D quantitative planar imaging

**Fig. 2:**
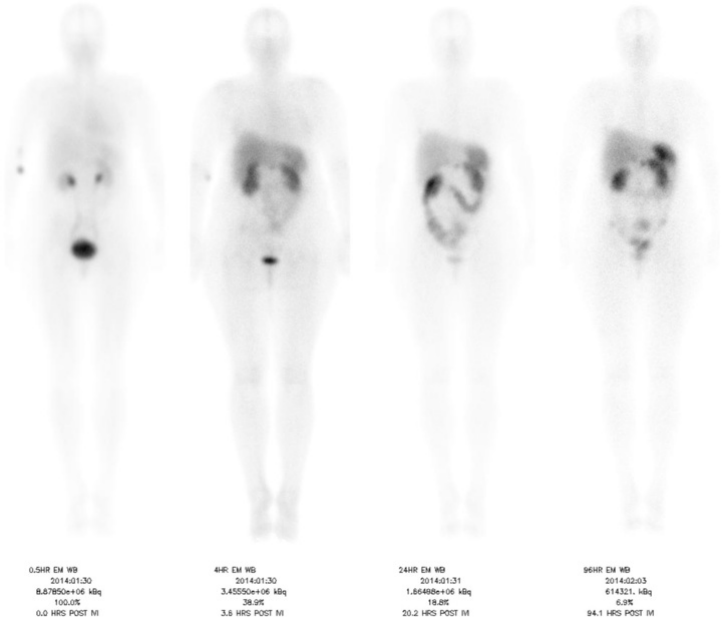
Whole-body quantitative planar scans for one subject (subject C in Table 3) for a single cycle from 0 to 96 h (approx.). The estimated retained radioactivity is estimated to be 8878, 3456, 1865 and 614 MBq for each of the time points, corresponding to 100, 39, 19 and 7 % retained, respectively. The calibration standard seen in Fig. 1 is removed by the software before the determination of the total radioactivity retained in the subject

Twenty-eight whole-body planar scans were acquired at the initial 30-min time point in nine individuals undergoing RNT, immediately after the completion of the [^177^Lu]-DOTATATE infusion. All subjects had previously been diagnosed with a histologically confirmed metastatic neuroendocrine tumour (NET), had demonstrated good uptake in the NET lesions on [^68^Ga]-DOTATATE PET/CT imaging and met all other inclusion criteria for RNT with [^177^Lu]-DOTATATE. An example of processed quantitative whole-body planar data is shown in Fig. 2 for all time points of a single cycle for one subject. Indicated in the text under each image is the estimated radioactivity in each scan and the fraction expressed as a percentage of the radioactivity in the first image, taken to be 100 %.

Table 3 shows the relevant subject characteristics and results for cohort 1, the dose calibrator measures of the administered amounts and the image-based estimated total radioactivity for the 26 initial whole-body planar scans. The estimated total body radioactivity of ^177^Lu had an average error of +4.6 ± 5.9 % with a range −17.1 to +11.2 %. Within an individual subject, the average difference between measured and estimated total radioactivity was in the range −4.0 to +7.9 %.

**Table 3:** Subject characteristics and results of planar whole-body radioactivity measurements for cohort 1. The height, weight and BMI (body mass index) are included to allow correlation between general measures of body habitus and the results from quantitative imaging

Identifier	Primary Dx	Height (cm)	Weight (kg)	BMI (kg/m^2^)	Cycle	Administered (MBq)	Measured (MBq)	Difference (%)	Subject average difference (%)
A	Small bowel NET	170	66	23	1	6232	6378	2.3	5.8
					2	8505	9327	9.7	
					3	7515	8215	9.3	
					4	7803	7956	2.0	
B	Pancreatic NET	177	88	28	1	6780	6494	−4.2	2.9
					2	8434	9110	8.0	
					3	8505	9060	6.5	
					4	7599	7708	1.4	
C	Breast NET	169	77	27	1	7985	7767	−2.7	4.2
					2	9096	9516	4.6	
					3	7996	8878	11.0	
					4	7821	8133	4.0	
D	Rectal NET	178	66	21	1	8711	9410	8.0	9.6
					2	7614	8465	11.2	
E	Small bowel NET	150	79	35	1	7971	8542	7.2	4.4
					2	7669	7689	0.3	
					3	7930	8392	5.8	
					4	8100	8012	−1.1	
F	Meningioma	180	88	27	1	7928	8730	10.1	7.9
					2	7560	8042	6.4	
					4	7900	8471	7.2	
G	Meningioma	168	68	24	1	7827	8529	9.0	6.0
					2	7391	7923	7.2	
					3	7897	8036	1.8	
H	Adrenal phaeochromocytoma	175	69	23	1	7618	8127	6.7	6.3
					2	7484	7925	5.9	
I	Pancreatic NET	183	93	28	1	7115	5898	−17.1	−4.0
					3	8160	8902	9.1	
	Mean	170.9	75.1	26.0		7841	8263	4.6	
							SD	5.9	

### 3D quantitative SPECT imaging

In cohort 2, 12 SPECT studies with blood samples taken during the early scan, acquired immediately after the end of the administration of the RNT, were acquired from six subjects. Table 4 contains all results for these measurements. There was a general tendency to *underestimate* the radioactivity concentration in the SPECT images of the blood pool using the previously defined experimental parameters for SPECT imaging of ^177^Lu on the particular gamma camera, collimator energy, PHA window and radionuclide used. The mean error was −4.0 ± 7.8 % with a range −16.1 to +7.5 %.

**Table 4:** Results comparing the whole blood and image-derived radioactivity concentration measurements for cohort 2

Identifier	Gamma counter blood activity (kBq mL^−1^)	SPECT average blood activity (kBq mL^−1^)	Difference (%)
1	317	298	−6.0
2	411	375	−8.8
3	404	349	−13.6
4	271	287	5.9
5	170	158	−7.1
6	150	142	−5.3
7	323	308	−4.8
8	302	315	4.3
9	301	274	−9.0
10	316	265	−16.1
11	360	387	7.5
12	235	246	4.7
		Mean	−4.0
		SD	7.8

**Fig. 3:**
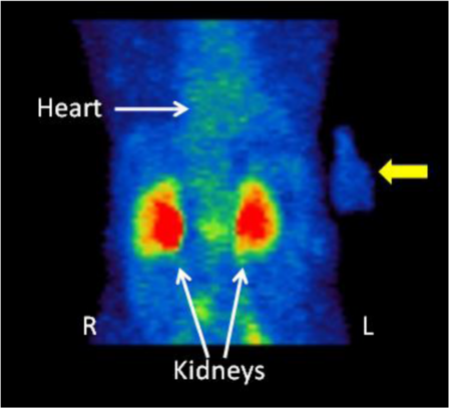
A MIP image from the initial SPECT study (0.5 h after administration of the therapy commenced) for one subject is shown, indicating the location of the calibration flask (*yellow arrow*) relative to the subject. The kidneys (*red*) are seen to be the organs with the highest uptake at this imaging time point. Subject’s right side (R) and left side (L) are indicated

We measured the total radioactivity in the calibration flask in 89 separate SPECT acquisitions from nine subjects each having up to four cycles of RNT—for each cycle, we have 3–4 SPECT acquisitions corresponding to the time points 0.5, 4, 24 and 96 or 120 h after administration (Table 4). An example of a maximum intensity projection (MIP) from a SPECT study at the 0.5-h time point showing the location of the calibration flask relative to the subject can be seen in Fig. 3. The average estimated difference between the radioactivity in the standard in the image and the amount dispensed as measured by the dose calibrator was +2.0 ± 8.5 % with a range of −16.2 to +24.2 %.

## Discussion

The recent introduction of a number of new theranostic radiopharmaceutical pairs has been an impetus for renewed interest in quantitative imaging with the gamma camera. These developments in synthetic radiochemistry have been accompanied by increasing utility of hybrid SPECT/CT gamma cameras and improvements in algorithms for quantitative image reconstruction. In this paper, we have re-introduced procedures documented more than 15 years ago [6] to produce quantitative 2D whole-body planar images for the therapeutic radionuclide ^177^Lu. In addition, we have applied techniques that we have previously developed for quantitative 3D SPECT imaging with ^99m^Tc and ^201^Tl [2] to ^177^Lu. While whole-body 3D quantitative SPECT imaging at multiple time points, such as available with PET, still remains challenging for SPECT, we are, nevertheless, able to characterise much of the biodistribution and time-course of radiopharmaceuticals labelled with ^177^Lu using current generation state-of-the-art multimodal gamma cameras.

Quantification of 2D planar whole-body imaging allows us to study the retention of the radiopharmaceutical in an individual subject. This is the first step towards estimating individual organ and tissue uptake. Absolute quantification is not essential for studying total body retention as this can be achieved by simply measuring the subject immediately after administering the radiopharmaceutical and calculating subsequent retention values as a fraction of the initial total count. The method implemented in this paper, being an absolute measure, relaxes this constraint, thus allowing whole-body estimates to be made at any time point without the initial “100 % of dose” image against which all other relative measures are based. The estimation of the radioactivity as the percentage of injected dose (%ID) in individual organs and tissues over time, however, requires a quantitative approach. Organ dosimetry based on 2D planar imaging has limitations due to the superimposition of different organs and tissues in the image and is therefore limited. However, such measurements are still generally acceptable when estimates of organ dosimetry are required. In time, it is likely that all radionuclide organ dosimetry estimates will be obtained with tomographic imaging, whether from PET or SPECT [12, 13], but currently, 2D planar whole-body imaging remains the standard. A potential problem that can arise when producing pixel-by-pixel geometric mean images from conjugate views, as opposed to calculating the geometric mean of two count rates from larger ROIs, is that in areas of low count rate a pixel containing a zero value may be encountered and thus will produce a geometric mean of 0. This would not happen with an arithmetic mean. The effect of this would be to *underestimate* the mean count rate and hence the radioactivity contained. This happens frequently if a background region outside the body is sampled. This underestimation is unlikely to be a problem with the [^177^Lu]-DOTATATE images we are using due to the large amounts of radioactivity administered in this therapeutic regime and subsequently the high total number of events that are recorded. In the scenario where ^177^Lu might be used as a tracer study prior to therapy, however, this could be a source of error.

We have shown in this paper that previously published methodology can be applied to planar whole-body imaging for ^177^Lu with sufficient accuracy for dosimetry calculations. Using the techniques described in this paper, we have recently reported on the biodistribution and radiation dosimetry from nca [^177^Lu]-DOTATATE [14], and this will be the subject of a future manuscript.

Quantitative SPECT imaging is a subject that has recently been receiving increasing attention [4, 15–18]. It is likely to increase even further as algorithms are developed with a primary focus on producing quantitative image data. Additionally, radionuclides that are used for therapeutic applications are generally more likely to produce gamma emissions than positrons and therefore will always be more suitable to imaging with the gamma camera/SPECT, or other single-photon imaging device, than with PET. One notable exception to this is the relatively recent introduction of imaging therapeutic ^90^Y distributions with the PET camera [19, 20]. Whole-body SPECT/CT imaging at multiple time points, while possible, still places excessive demands on the individuals being imaged due to the lengthy acquisition times and hence is not commonly used today. We have combined whole-body planar imaging with regional SPECT/CT imaging over organs and tissues of interest at multiple time points to better characterise the delivery and retention of radionuclide therapy with [^177^Lu]-DOTATATE.

In this paper, we have demonstrated that quantitative imaging with the gamma camera using whole-body planar and SPECT/CT methods can produce images accurate to better than ±10 % on average of the true value. The planar image tended to overestimate the true radioactivity amount (by ~5 % on average) while the SPECT studies tended to underestimate the radioactivity concentration by a similar amount (−4 % on average). The check of the internal standard in SPECT was, in general, extremely accurate. In two of the subjects, the radioactivity concentrations were underestimated by >10 % (subject identifier #3: −13.6 % and subject identifier #10: −16.1 %). In fact, the results for subjects with identifiers 1, 2 and 3 are the same individual measured during different cycles, and the same is also true for subject identifiers 7, 8, 9 and 10 (in a different individual). As can be seen in Table 3, the larger error seen for identifier 3 is worse than for identifiers 1 and 2, and likewise the error in estimation for identifiers 7, 8, and 9 are less than that seen for identifier 10. A check of the accuracy of the internal standard in the study with identifier 3 reveals an error of −2.2 %, and for identifier 10, the error in the internal standard was +1.7 %. Therefore, the larger errors seen when measuring the blood pool radioactivity concentration are unlikely to be systematic errors due to a common problem (e.g. global dead time) and are more likely related to issues in obtaining the sample. As mentioned previously, the rate of change of the radioactivity concentration is very great in the first few hours after administration of [^177^Lu]-DOTATATE in subjects with good renal function. However, in general, the values obtained are likely to be acceptable for most quantitative work in nuclear medicine imaging, particularly radiation dosimetry estimation, given the other factors that contribute to the derivation of such estimates.

These results with ^177^Lu are consistent with previous publications that have tested the accuracy of SPECT reconstructions using diagnostic imaging radionuclides such as ^99m^Tc, ^123^I and ^201^Tl [2, 7–9, 15, 16, 21–24].

We have used the quantitative SPECT data to estimate the radiation-absorbed dose to the kidneys, which is seen as the organ most at risk, with each cycle of [^177^Lu]-DOTATATE therapy. This paper reports on the accuracy of in vivo measurements in human subjects, rather than experimental phantom studies, and as such demonstrates the ability of the imaging techniques to provide meaningful quantitative data in clinically relevant situations. We are also able to estimate the radiation absorbed dose to any neoplastic lesions in the same field of view; however, other lesions throughout the body have not generally been imaged due to the need for additional SPECT acquisitions to cover these regions. We envisage that improvements in reconstruction algorithms may lead to a reduction in acquisition times which then may be used to study multiple regions of the body in the same total scan time as is currently required.

We have used internal standards of the same radionuclide for both the planar whole-body and SPECT measurements extensively in these studies. We consider that this provides useful additional information plus internal quality assurance for any absolute quantification regime. In the planar whole-body studies, the standard is used to (a) check the sensitivity (cps kBq^−1^) in whole-body scanning mode, which varies with changes in the scanning bed speed, as well as (b) assessing that the total number of events measured in the flask decreases with time due to radioactive decay which can be used to verify that the gamma camera is behaving in a linear fashion and is not being unduly compromised by, for example, high dead time. In the SPECT studies, the calibration standard is used (i) to check the accuracy of the recovered total radioactivity in the standard in the reconstructed images and (ii) to check that the reconstructed total radioactivity decreases over time again due to radioactive decay and produces a good estimate of the physical half-life of the radionuclide in question.

## Conclusions

 The quantitative data that we have produced with these techniques can be used to estimate a variety of parameters of interest in radionuclide therapy including total body retained radioactivity (for radiation safety purposes), planar MIRD-based internal radionuclide dosimetry, 3D estimates of radiation dosimetry per cycle to target tissues (neoplasia) and to organs at risk (e.g. kidneys), and to monitor changes over time, for example, changes in uptake in response to the therapy. As we now regard SPECT as a quantitative tool, further investigations that were not previously possible can now be undertaken such as examining the potential impact of concomitant “modulating” or “blocking” pharmaceuticals on therapeutic radiopharmaceutical uptake.
